# Supplementary material for: Establishing flood thresholds for sea level rise impact communication
Source: Nat Commun. 2024 May 18;15:4251. doi: 10.1038/s41467-024-48545-1 (PMC11102483; doi:10.1038/s41467-024-48545-1)
Supplement: Supplementary file 1 — Supplementary Information [file 41467_2024_48545_MOESM1_ESM.pdf]

Supplementary Information for

Establishing flood thresholds for sea level rise impact communication

Sadaf Mahmoudi[1,2*], Hamed Moftakhari[1,2*], David F. Muñoz[3], William Sweet[4], Hamid Moradkhani[1,2]

1. Center for Complex Hydrosystems Research, The University of Alabama, Tuscaloosa, AL, USA
2. Department of Civil, Construction, and Environmental Engineering, The University of Alabama, Tuscaloosa, AL, USA
3. Department of Civil and Environmental Engineering, Virginia Tech, Blacksburg, VA, USA
4. NOAA/National Ocean Service, Silver Spring, MD, USA

*Corresponding authors: smahmoudikouhi@crimson.ua.edu; hmoftakhari@ua.edu

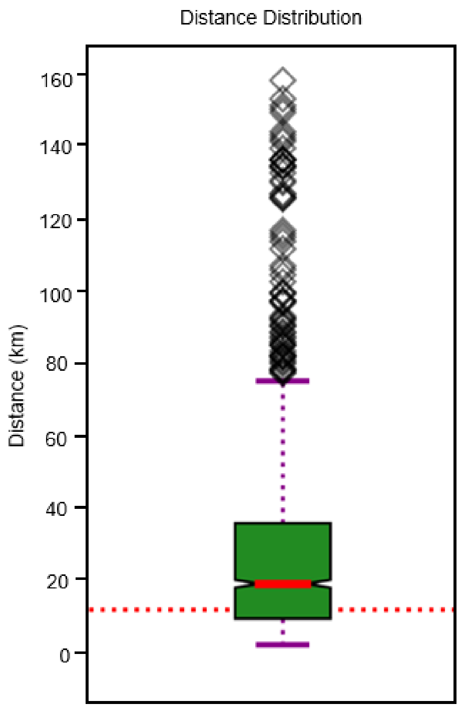

Figure S1. The boxplot of the distance between US coastal communities and their nearest tide gauge

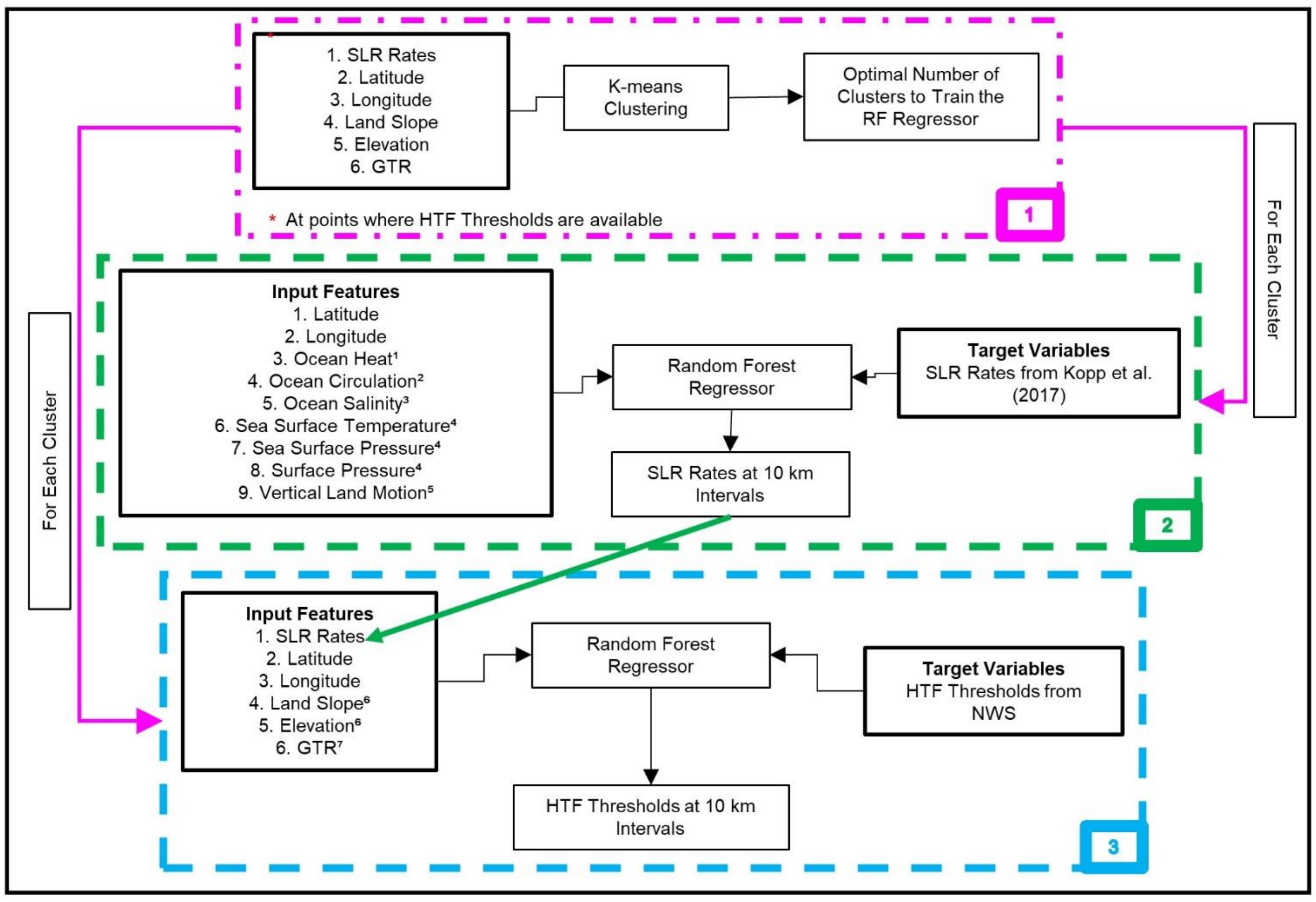

Figure S2. Flowchart of this study

Table S1. Characteristics of the input features used in ML algorithms.

Name of Dataset	Resolution	Reference	Unit	Dataset Title	URL
[1]Global Ocean Heat Content	1°×1°	Improved estimates of ocean heat content from 1960 to 2015	J/m^2	IAP 2-D Ocean Heat Content using IAP T/S data	http://www.ocean.iap.ac.cn/pages/dataService/dataService.html?languageType=en&navAnchor=dataService
[2]Ocean Circulation		Diagnostic Model and Analysis of the Surface Currents in the Tropical Pacific Ocean	m/s	OSCAR 1 degree ocean surface currents	https://podaac-tools.jpl.nasa.gov/drive/files/allData/oscar/L4/oscar_1_deg
[3]Salinity	1°×1°	Provided by the NOAA PSL, Boulder, Colorado, USA, from their website at https://psl.noaa.gov	kg/kg	GODAS: Global Ocean Data Assimilation System	https://downloads.psl.noaa.gov/Datasets/godas/
[4]Sea level Pressure and Surface Pressure	1°×1°	NOAA National Centers for Environmental Information (NCEI)	millibars	International Comprehensive Ocean-Atmosphere Data Set	https://cds.climate.copernicus.eu/cdsapp#!/dataset/reanalysis-era5-single-levels-monthly-means?tab=form
[4]Sea Surface Temperature	1°×1°	NOAA National Centers for Environmental Information (NCEI)	degC	International Comprehensive Ocean-Atmosphere Data Set (ICOADS)	https://cds.climate.copernicus.eu/cdsapp#!/dataset/reanalysis-era5-single-levels-monthly-means?tab=form
[5]Vertical Land Motion		A new global GPS dataset for testing and improving modeled GIA uplift rates	mm/a	Vertical Land Motion (VLM)	https://doi.pangaea.de/10.1594/PANGAEA.889923?format=textfile
[6]Greater Tide Range	10 Km	White, S. A. (2013). VDatum: Vertical Datum Transformation Tool. Presented to the Hydrographic Services Review Panel.	m	Vertical Datum Transformation	https://vdatum.noaa.gov/vdatumweb/
[7]Land Slope	15 arc-second	Derived by GEBCO DEMs (GEBCO Compilation Group (2022) GEBCO_2022 Grid (doi:10.5285/e0f0bb80-ab44-2739-e053-6c86abc0289c)_	degree	-	https://www.gebco.net/data_and_products/gridded_bathymetry_data/
[7]Elevation	15 arc-second	Derived by GEBCO DEMs (GEBCO Compilation Group (2022) GEBCO_2022 Grid (doi:10.5285/e0f0bb80-ab44-2739-e053-6c86abc0289c)_	degree	-	https://www.gebco.net/data_and_products/gridded_bathymetry_data/

Table S2. Parameters of SLR ML algorithms.

SLR Rates			
		number of estimators	maximum depth
RCP 4.5, 5th Percentile	West Coast	140	10
	Gulf/SE Coasts	100	10
	NE Coast	140	10
RCP 4.5, 50th Percentile	West Coast	120	10
	Gulf/SE Coasts	90	9
	NE Coast	140	10
RCP 4.5, 95th Percentile	West Coast	150	10
	Gulf/SE Coasts	160	10
	NE Coast	160	10
RCP 8.5, 5th Percentile	West Coast	130	10
	Gulf/SE Coasts	100	9
	NE Coast	140	10
RCP 8.5, 50th Percentile	West Coast	140	10
	Gulf/SE Coasts	100	9
	NE Coast	140	10
RCP 8.5, 95th Percentile	West Coast	120	10
	Gulf/SE Coasts	100	9
	NE Coast	130	9

Table S3. Evaluation metrics of SLR ML algorithms under different scenarios.

		RCP 4.5				RCP 8.5			
percentile	Cluster	MAE	R Squared	NSE	KGE	MAE	R Squared	NSE	KGE
5	First	0.29	0.92	0.92	0.86	0.16	0.94	0.94	0.9
	Second	0.1	1	1	0.97	0.1	0.99	0.99	0.97
	Third	0.05	0.99	0.99	0.98	0.11	0.97	0.97	0.96
50	First	0.17	0.95	0.95	0.93	0.11	0.97	0.97	0.95
	Second	0.11	0.99	0.99	0.97	0.1	0.99	0.99	0.97
	Third	0.08	0.97	0.97	0.96	0.09	0.97	0.97	0.95
95	First	0.16	0.96	0.96	0.94	0.1	0.98	0.98	0.96
	Second	0.13	0.99	0.99	0.97	0.11	0.99	0.99	0.97
	Third	0.08	0.96	0.96	0.95	0.11	0.96	0.96	0.95

Table S4. Feature importance of SLR ML algorithms under different scenarios.

		RCP 4.5								
percentile	Cluster	Latitude	Longitude	Global Heat	Ocean Circulation	Salinity	Sea surface Pressure	Surface Pressure	Sea Surface Temperature	Vertical Land Motion
5	First	0.21	0.07	0.07	0.25	0.21	0.04	0.03	0.03	0.08
	Second	0.01	0.11	0.04	0.8	0	0	0	0.02	0.01
	Third	0.58	0.01	0.05	0.02	0.08	0.06	0.06	0.07	0.06
50	First	0.25	0.1	0.11	0.11	0.16	0.05	0.05	0.02	0.14
	Second	0.01	0.09	0.05	0.79	0.04	0	0	0.02	0.01
	Third	0.29	0.02	0.09	0.02	0.04	0.04	0.13	0.12	0.25
95	First	0.28	0.09	0.11	0.13	0.12	0.06	0.06	0.02	0.14
	Second	0.01	0.09	0.04	0.8	0.04	0	0	0.02	0.01
	Third	0.25	0.02	0.09	0.04	0.1	0.03	0.27	0.03	0.18

		RCP 8.5								
percentile	Cluster	Latitude	Longitude	Global Heat	Ocean Circulation	Salinity	Sea surface Pressure	Surface Pressure	Sea Surface Temperature	Vertical Land Motion
5	First	0.25	0.11	0.1	0.17	0.11	0.04	0.04	0.04	0.14
	Second	0.01	0.09	0.05	0.77	0.02	0	0	0.03	0.01
	Third	0.31	0.07	0.02	0.02	0.36	0.01	0.13	0.03	0.04
50	First	0.18	0.17	0.12	0.12	0.13	0.03	0.04	0.01	0.19
	Second	0.01	0.09	0.03	0.81	0.03	0	0	0.01	0.01
	Third	0.49	0.05	0.1	0.03	0.08	0.03	0.07	0.03	0.1
95	First	0.25	0.12	0.1	0.14	0.13	0.04	0.05	0.02	0.16
	Second	0	0.09	0.04	0.82	0.02	0	0	0.01	0
	Third	0.22	0.23	0.08	0.01	0.05	0.05	0.06	0.05	0.24

Table S5. Parameters of HTF ML algorithms.

HTF Thresholds			
		number of estimators	maximum depth
RCP 4.5, 5th Percentile	West Coast	140	7
	Gulf/SE Coasts	130	10
	NE Coast	130	9
RCP 4.5, 50th Percentile	West Coast	150	7
	Gulf/SE Coasts	150	10
	NE Coast	160	9
RCP 4.5, 95th Percentile	West Coast	140	7
	Gulf/SE Coasts	100	10
	NE Coast	170	9
RCP 8.5, 5th Percentile	West Coast	130	7
	Gulf/SE Coasts	170	10
	NE Coast	130	10
RCP 8.5, 50th Percentile	West Coast	110	7
	Gulf/SE Coasts	170	10
	NE Coast	160	9
RCP 8.5, 95th Percentile	West Coast	140	7
	Gulf/SE Coasts	160	10
	NE Coast	140	10

Table S6. Evaluation metrics of HTF ML algorithms under different scenarios; a) with considering coastal elevation and continental shelf slope, b) without considering coastal elevation and continental shelf slope

a)		RCP 4.5				RCP 8.5			
percentile	Cluster	MAE	R Squared	NSE	KGE	MAE	R Squared	NSE	KGE
5	First	0.02	0.71	0.69	0.74	0.02	0.69	0.66	0.74
	Second	0.07	0.59	0.56	0.66	0.06	0.59	0.56	0.69
	Third	0.08	0.52	0.45	0.68	0.09	0.44	0.31	0.61
50	First	0.03	0.67	0.64	0.69	0.02	0.71	0.68	0.72
	Second	0.07	0.58	0.56	0.67	0.07	0.57	0.54	0.67
	Third	0.09	0.48	0.41	0.62	0.09	0.49	0.39	0.64
95	First	0.03	0.68	0.65	0.69	0.02	0.69	0.66	0.71
	Second	0.07	0.55	0.52	0.68	0.07	0.55	0.51	0.66
	Third	0.09	0.47	0.39	0.62	0.08	0.5	0.43	0.65
b)		RCP 4.5				RCP 8.5			
percentile	Cluster	MAE	R Squared	NSE	KGE	MAE	R Squared	NSE	KGE
5	First	0.02	0.79	0.77	0.83	0.01	0.82	0.8	0.87
	Second	0.06	0.63	0.59	0.72	0.06	0.66	0.63	0.75
	Third	0.07	0.59	0.54	0.72	0.08	0.52	0.42	0.68
50	First	0.01	0.86	0.85	0.89	0.01	0.82	0.79	0.85
	Second	0.06	0.64	0.6	0.73	0.06	0.67	0.64	0.73
	Third	0.07	0.54	0.48	0.68	0.07	0.57	0.51	0.71
95	First	0.01	0.82	0.8	0.84	0.01	0.85	0.83	0.87
	Second	0.06	0.62	0.56	0.72	0.06	0.62	0.58	0.73
	Third	0.07	0.56	0.51	0.69	0.07	0.56	0.48	0.7

Table S7. Feature importance of HTF ML algorithms under different scenarios.

		RCP 4.5					
percentile	Cluster	Latitude	Longitude	Tidal Range	Sea Level Rise	Continental Shelf Slope	Coastal Elevation
5	First	0.39	0.19	0.15	0.19	0.03	0.05
	Second	0.18	0.5	0.09	0.1	0.06	0.07
	Third	0.26	0.23	0.24	0.17	0.05	0.04
50	First	0.38	0.21	0.16	0.17	0.03	0.05
	Second	0.17	0.5	0.08	0.12	0.06	0.07
	Third	0.29	0.21	0.25	0.15	0.06	0.05
95	First	0.49	0.22	0.17	0.04	0.03	0.05
	Second	0.17	0.51	0.09	0.1	0.06	0.07
	Third	0.29	0.22	0.24	0.15	0.06	0.05

		RCP 8.5					
percentile	Cluster	Latitude	Longitude	Tidal Range	Sea Level Rise	Continental Shelf Slope	Coastal Elevation
5	First	0.38	0.2	0.16	0.18	0.03	0.05
	Second	0.17	0.49	0.1	0.11	0.06	0.07
	Third	0.24	0.16	0.18	0.28	0.07	0.05
50	First	0.38	0.21	0.16	0.17	0.03	0.05
	Second	0.17	0.5	0.1	0.1	0.06	0.07
	Third	0.28	0.22	0.24	0.16	0.06	0.04
95	First	0.4	0.21	0.15	0.18	0.02	0.04
	Second	0.17	0.5	0.09	0.1	0.06	0.07
	Third	0.3	0.22	0.25	0.12	0.07	0.05

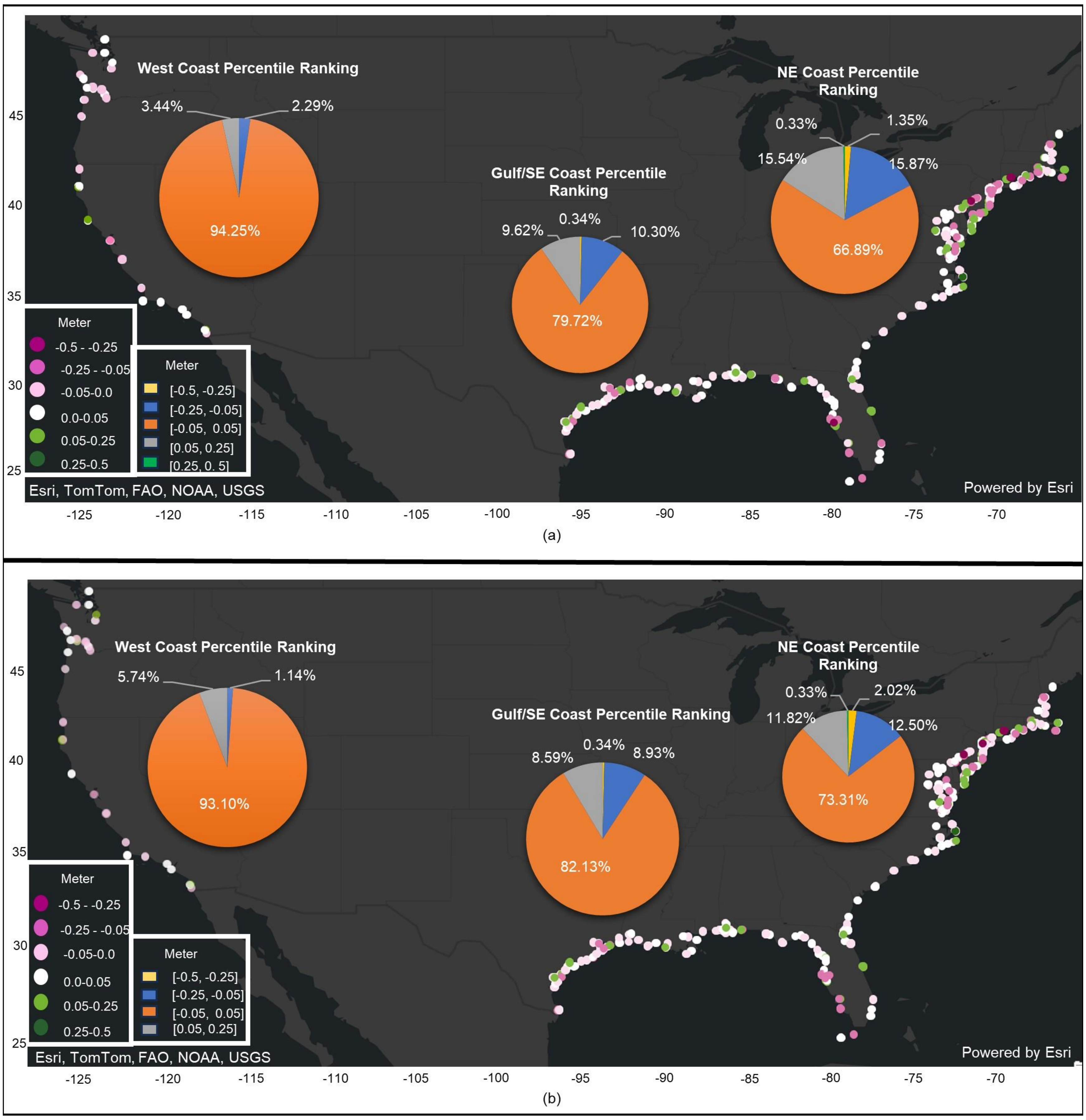

Figure S3. Percentile ranking of the difference between the official and ML-predicted thresholds a) with the coastal elevation and continental shelf slope, b) without the coastal elevation and continental shelf slope as input features. The points show the amount of difference.

Table S8. The average validation evaluation metrics of a) SLR Rates, b) HTF Thresholds with elevation and slope, c) HTF Thresholds without elevation and slope

a)

		RCP 4.5				RCP 8.5			
percentile	Cluster	MAE	R Squared	NSE	KGE	MAE	R Squared	NSE	KGE
5	First	0.63	0.76	0.69	0.74	0.52	0.77	0.75	0.77
	Second	0.37	0.94	0.93	0.92	0.38	0.94	0.92	0.9
	Third	0.23	0.9	0.89	0.91	0.41	0.89	0.86	0.85
50	First	0.54	0.77	0.74	0.82	0.54	0.72	0.72	0.81
	Second	0.32	0.95	0.95	0.94	0.27	0.97	0.96	0.95
	Third	0.29	0.85	0.8	0.85	0.25	0.86	0.82	0.86
95	First	0.59	0.76	0.72	0.82	0.56	0.74	0.74	0.82
	Second	0.3	0.95	0.94	0.94	0.26	0.96	0.96	0.96
	Third	0.25	0.86	0.85	0.89	0.46	0.77	0.69	0.78

b)

		RCP 4.5				RCP 8.5			
percentile	Cluster	MAE	R Squared	NSE	KGE	MAE	R Squared	NSE	KGE
5	First	0.05	0.43	0.33	0.5	0.05	0.45	0.35	0.52
	Second	0.08	0.38	0.35	0.5	0.09	0.37	0.31	0.5
	Third	0.14	0.34	0.28	0.45	0.15	0.3	0.2	0.45
50	First	0.06	0.47	0.36	0.5	0.05	0.44	0.33	0.5
	Second	0.08	0.39	0.33	0.51	0.08	0.4	0.32	0.5
	Third	0.13	0.33	0.26	0.44	0.14	0.34	0.25	0.45
95	First	0.05	0.46	0.34	0.52	0.05	0.46	0.34	0.54
	Second	0.08	0.4	0.34	0.5	0.08	0.39	0.33	0.5
	Third	0.13	0.3	0.25	0.4	0.14	0.29	0.21	0.4

c)

		RCP 4.5				RCP 8.5			
percentile	Cluster	MAE	R Squared	NSE	KGE	MAE	R Squared	NSE	KGE
5	First	0.05	0.49	0.38	0.59	0.05	0.51	0.39	0.6
	Second	0.09	0.43	0.38	0.51	0.09	0.41	0.34	0.51
	Third	0.13	0.35	0.28	0.46	0.15	0.31	0.19	0.44
50	First	0.04	0.52	0.42	0.6	0.05	0.5	0.38	0.58
	Second	0.08	0.44	0.4	0.54	0.08	0.44	0.38	0.54
	Third	0.13	0.36	0.27	0.48	0.12	0.39	0.3	0.49
95	First	0.05	0.5	0.39	0.6	0.05	0.5	0.38	0.6
	Second	0.08	0.42	0.36	0.54	0.08	0.43	0.38	0.53
	Third	0.13	0.33	0.24	0.44	0.14	0.3	0.21	0.4
